# Using Macro-Arrays to Study Routes of Infection of *Helicobacter pylori* in Three Families

**DOI:** 10.1371/journal.pone.0002259

**Published:** 2008-05-21

**Authors:** Josette Raymond, Jean-Michel Thiberge, Nicolas Kalach, Michel Bergeret, Christophe Dupont, Agnès Labigne, Catherine Dauga

**Affiliations:** 1 Service de Bactériologie, Université Paris V, Hôpital Cochin, Paris, France; 2 Unité de Pathogénie Bactérienne des Muqueuses, Département de Pathogénie Microbienne, Paris, France; 3 Service de Pédiatrie, Hôpital Saint Antoine, Pédiatrie, Lille, France; 4 Département de Pédiatrie, Hôpital Cochin-Saint Vincent de Paul, Paris, France; 5 Plateforme 4—Génopole, Département Génétique des Génomes, Institut Pasteur, Paris, France; New York University, United States of America

## Abstract

**Background:**

Analysis of the evolutionary dynamics of *Helicobacter pylori* allowed tracing the spread of infection through populations on different continents but transmission pathways between individual humans have not been clearly described.

**Materials and Methods:**

To investigate person-to-person transmission, we studied three families each including one child with persistence of symptoms after antibiotic treatment. Ten isolates from the antrum and corpus of stomach of each family member were analyzed both by sequencing of two housekeeping genes and macroarray tests.

**Results:**

A total of 134 (8.4%) out of the 1590 coding sequences (CDSs) tested, including *cag* PAI and insertion sequences, were present in some but not all isolates (and are therefore defined as variable CDSs). Most of the variable CDSs encoded proteins of unknown function (76/134) or were selfish DNA including that encoding restriction/modification enzymes (13/134). Isolates colonizing the stomach of one individual can vary by point mutations, as seen in *hspA*, or by the gain or loss of one to five CDSs. They were considered as (genetic) variants. The phylogenetic clustering of gene profiles obtained on macro-arrays allowed identifying the different strains infecting families. Two to five strains circulated within a family. Identical strains were present in at least two members of all three families supporting the accepted model of intrafamilial transmission. Surprisingly, the mother was not implicated in the transmission of *H. pylori* in the two French families. Sibling-to-sibling transmission and acquisition of *H. pylori* from outside the family appeared to be probable in the transmission pathways.

**Conclusion:**

Macroarray analysis based on previously selected CDSs gives a comprehensive view of the genome diversity of a pathogen. This approach combined with information on the origin of the *hspA* and *glmM* alleles revealed that *Helicobacter pylori* infection may be acquired by more diverse routes than previously expected.

## Introduction

*Helicobacter pylori* is the cause of several gastroduodenal diseases, including chronic gastritis, peptic ulcer and gastric carcinoma [1]–[3]. *H. pylori* strains appear spread by person-to-person contact and humans seem the only identified source of infection [4]. Prevalence studies suggest that infection is mostly acquired during childhood, and parent-to-child infection, especially involving infected mothers, has been suggested to be the major route of transmission [5], [6]. However, evidence for sibling transmission has been also reported and the exact routes of transmission remain elusive [7].

Familial transmission of infection has been investigated by molecular typing studies, identifying clones shared by family members [8]–[11]. Clonal descent among the *H. pylori* isolates infecting a family has been documented in one isolate from each biopsy of different members of families by comparing alleles of genes including *vacA*, *flaA* and *flaB* [12] and by sequencing three housekeeping genes (*ureI, atpA* and *ahpC*) [13]. Phylogenetic analysis assessing the history of genes revealed frequent recombination for several genes in *H. pylori* [14]–[16]. Indeed, homologous recombination, highly dependent on sequence similarity, is expected to be frequent within this species [17]. Therefore, it is important for epidemiological analysis, not only to study the transmission of a small number of genes between isolates from different members of a family, but also to investigate their whole genomic diversity.

The genetic diversity and evolutionary dynamics of *H. pylori* isolates strains can be explored by using macro-arrays to examine strain-specific genes. The whole genomes of *H. pylori* strains J99 (isolated from a white American in Tennessee with ulcer and belonging to the hspWAfrica subpopulation of hpAfrica1 [18] and 26695 (isolated from a patient from United Kingdom with gastritis belonging to the hpEurope population) have been compared revealing regions that have extremely variable gene content that are referred to as “plasticity zones” (PZ)[19]. They are the preferred sites for the insertion of a pathogenicity island *cag*, *cag*PAI, which is a large mobile element associated with an enhanced risk for the development of duodenal ulcers and adenocarcinoma of the distal stomach [20]. Also, several restriction/modification genes (R-M) often associated with insertion or repeat elements differ in terms of GC content from the rest of the genome; these genes may aid the bacteria under particular circumstances during their long-term infection of genetically diverse hosts [18]. The analysis of the presence or absence of genes at such loci gives an overall indication of diversity and can help to distinguish strains in various clinical contexts [20], [21].

We previously studied intrafamilial spread by analysing polymorphism of two housekeeping genes (*hspA* and *glmM*) following the failure of treatment to eradicate infection of a child [22]. Here, in addition to sequence analysis, we investigated the whole genome composition in *H. pylori* isolates colonizing the stomachs of infected members of three different families. Macro-arrays were used to test for 248 non ubiquitous open reading frames (ORFs) and 48 ubiquitous ORFs. Gene profiles were used to reconstruct gene transfer and gene loss events that had occurred in isolates. Phylogenetic analysis based on housekeeping genes sequences and genome composition in non ubiquitous genes provided an overview of the evolutionary dynamics of *H. pylori* strains infecting each family. Isolates with slight differences in gene content belonging to a same strain were carried by more than one family member, suggesting that circulation of strains between different hosts makes a significant contribution to the genomic diversity of *H. pylori*. Furthermore, re-infection of a child with a strain, with a genetic profile never observed in the family (before and after treatment*)* suggests that *Helicobacter pylori* can be acquired from outside the family.

## Results

**Figure 1:**
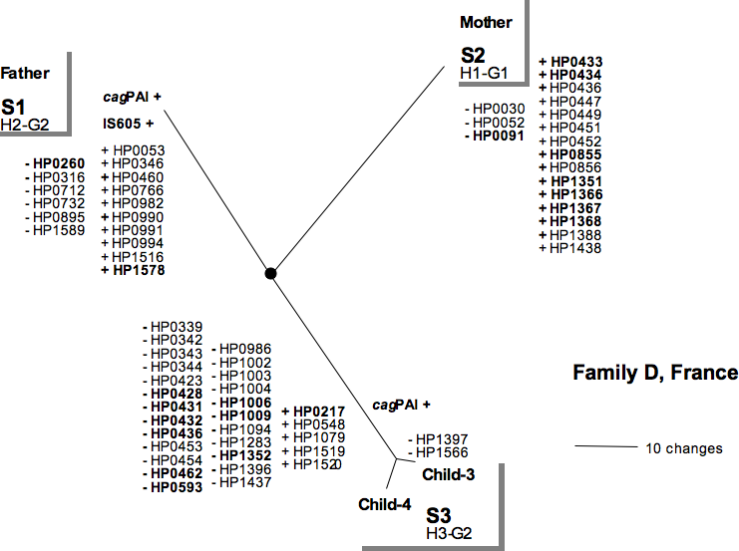
Parsimony analysis of macroarray data for isolates from family D. Strain numbers and genotypes are indicated. Absence (−) or presence (+) of CDS considered as character changes in the parsimony analysis are given for each node and peripheral branch. IS605 and *cag*PAI are shown when present. Names in bold indicate CDSs of known function (Table 1).

**Figure 2:**
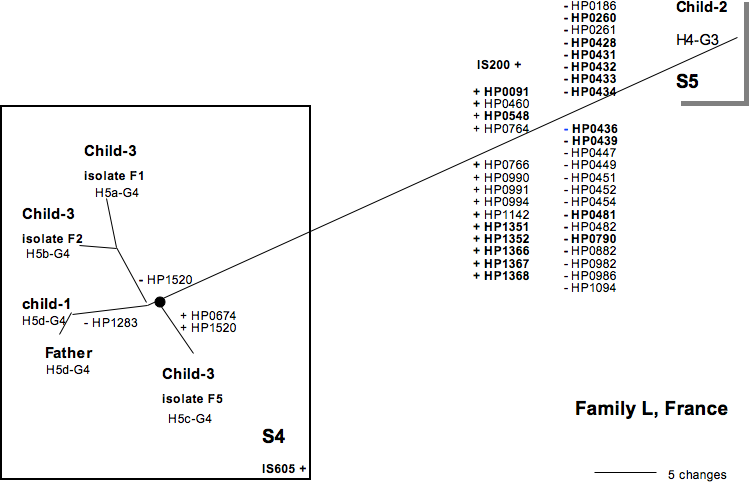
Parsimony analysis of macroarray data for isolates from family L. Strain numbers and genotypes are indicated. Absence (−) or presence (+) of CDS considered as character changes in the parsimony analysis are given for each node and peripheral branch. IS605 is shown when present. Names in bold correspond to CDSs of known function (Table 1).

**Figure 3:**
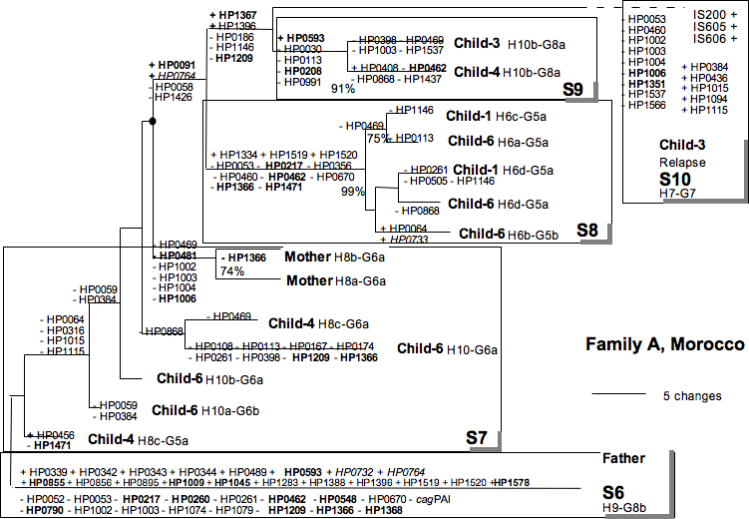
Parsimony analysis of macroarray data for isolates from family A. Bootstrap values above 70% are indicated at each node. Strain numbers and genotypes are indicated. Five strains (S6 to S10) were individualized. Strain S6 and S10 were clearly different from the other strains, according to their CDS content and their *hspA* and *glmM* alleles. Absence (−) or presence (+) of CDS considered as character changes in the parsimonious analysis are given for each node and peripheral branch. IS are shown when present. *cag*PAI is present in all the strains, except strain S6 from the father. Names in bold indicate CDSs of known function (Table 1). Names in italics indicate remnant genes.

We compared the gene content of isolates circulating in three infected families with the genes representative of the genomic diversity of 132 strains isolated from patients suffered from various clinical diseases in Africa, Asia and Europe (41 genes always present and 213 genes variably present in genomes). A cladistic analysis based on gene content of isolates allowed strains circulating in each infected family to be defined (Fig 1, 2, 3). Each isolate of infected families was a genetic variant with its own gene content. Each strain harbored a specific gene profile.

### Gene Content and functions of variably present genes in family isolates

Macro-arrays experiments showed that 134 of the 254 investigated coding sequences (CDSs) were present in some but not all of the 26 isolates from the three families. Among them,17 genes variably present in isolates from family A, seem being previously acquired by horizontal gene transfer from other species according to their atypical dinucleotide composition [23]. Fifteen variable CDSs in isolates from family D and 3 in those from family L corresponded also to these apparently foreign genes.

Genes within *cag*PAI (22 CDSs tested), IS200, IS605 and IS606 (11 CDSs), were absent from numerous isolates. Most of the variable CDSs encoded proteins of unknown function (76/134 CDSs) or were selfish DNA, such as that encoding restriction/modification enzymes (13 CDSs). Six genes (HP0428, HP0431, HP0432, HP0433, HP0434 and HP0436), not present in the genome of the strain J99 were variably present in isolates. Furthermore, two genes involved in DNA transfer (HP0525 and HP1006), two genes involved in lipopolysaccharides synthesis (HP0208 and HP1578), two CDSs belonging to the bacterial metabolism (coding for an alginate O-acetylation protein (*algI*, HP0855) and an acetyl-CoA synthetase (*acoE*, HP1045), were variably present (Table 1).

**Table 1:** List and function of genes studied with macroarrays.

Gene ID	Annotation of the genome of *H. pylori* 26695
***HP0026+***	**citrate synthase (gltA)**
*HP0030*	hypothetical protein
*HP0031*	hypothetical protein
*HP0035*	hypothetical protein
***HP0051***	**cytosine specific DNA methyltransferase (DDEM)**
*HP0052*	hypothetical protein
*HP0053*	hypothetical protein
***HP0054***	**adenine/cytosine DNA methyltransferase**
*HP0058*	hypothetical protein+frameshift
*HP0059*	hypothetical protein
*HP0063*	hypothetical protein
*HP0064*	hypothetical protein
*HP0065*	hypothetical protein
***HP0066***	**conserved hypothetical ATP-binding protein**
***HP0079***	**outer membrane protein (omp3)**
***HP0091***	**type II restriction enzyme R protein (hsdR)**
*HP0101*	hypothetical protein
*HP0108*	hypothetical protein
*HP0113+*	hypothetical protein
*HP0129*	hypothetical protein
*HP0167*	hypothetical protein
*HP0174*	hypothetical protein
*HP0186*	hypothetical protein
***HP0197+***	**S-adenosylmethionine synthetase 2 (metX)**
*HP0205*	hypothetical protein
*HP0208*	**lipopolysaccharide 1,2-glucosyltransferase (rfaJ) (frameshift)**
*HP0217*	**cgtA,β 1–4 N acetyl galactosamine transferase**
*HP0236*	hypothetical protein
*HP0241*	hypothetical protein
***HP0246+***	**flagellar basal-body P-ring protein (flgI)**
*HP0256*	hypothetical protein
***HP0260***	**adenine specific DNA methyltransferase (mod)**
*HP0261*	hypothetical protein
*HP0273*	hypothetical protein
*HP0287*	hypothetical protein
*HP0311*	hypothetical protein
*HP0316*	hypothetical protein
***HP0327***	**flagellar protein G (flaG)**
*HP0336*	**Cystin Rich protein, (** ***hcpB*** **)**
*HP0338*	hypothetical protein
*HP0339*	hypothetical protein
*HP0342*	hypothetical protein
*HP0343*	hypothetical protein
*HP0344*	hypothetical protein
*HP0346*	hypothetical protein
*HP0350*	hypothetical protein
*HP0356*	hypothetical protein
*HP0367*	hypothetical protein
*HP0368*	**Restriction modification system S subunit**
*HP0369*	**Methyltransferase**
***HP0372***	**deoxycytidine triphosphate deaminase (dcd)**
*HP0373*	**Putative outer membrane protein (** ***homC*** **)**
*HP0374*	hypothetical protein
***HP0377***	**thiol:disulfide interchange protein (dsbC), putative**
*HP0383*	hypothetical protein
*HP0384*	hypothetical protein
***HP0391***	**purine-binding chemotaxis protein (cheW)**
*HP0398*	hypothetical protein
***HP0402+***	**phenylalanyl-tRNA synthetase, beta subunit (pheT)**
*HP0408*	hypothetical protein
***HP0413***	**transposase-like protein, PS3IS**
***HP0414***	**IS200 insertion sequence from SARA17**
*HP0423*	hypothetical protein
*HP0424*	hypothetical protein
*HP0425*	hypothetical protein
*HP0426*	hypothetical protein
***HP0428***	**phage/colicin/tellurite resistance cluster terY protein**
***HP0431***	**protein phosphatase 2C homolog (ptc1)**
***HP0432***	**protein kinase C-like protein**
*HP0433*	hypothetical protein
*HP0434*	hypothetical protein
*HP0436*	hypothetical protein
***HP0437***	**IS605 transposase (tnpA)**
***HP0438***	**IS605 transposase (tnpB)**
*HP0439*	hypothetical protein
***HP0441***	**VirB4 homolog = DNA transfer protein**
*HP0444*	hypothetical protein
*HP0446*	hypothetical protein
*HP0447*	hypothetical protein
*HP0449*	hypothetical protein
*HP0451*	hypothetical protein
*HP0452*	hypothetical protein
*HP0453*	hypothetical protein
*HP0454*	hypothetical protein
*HP0456*	hypothetical protein
***HP0459***	**virB4 homolog (virB4)**
*HP0460*	hypothetical protein
***HP0462***	**type I restriction enzyme S protein (hsdS)**
*HP0469*	hypothetical protein
***HP0473***	**molybdenum ABC transporter, periplasmic molybdate-binding protein (modA)**
***HP0474+***	**molybdenum ABC transporter, permease protein (modB)**
***HP0475+***	**molybdenum ABC transporter, ATP-binding protein (modD)**
*HP0479*	**non functional restriction-modification**
***HP0481***	**Adenine-specific DNA methyltransferase (MFOKI)**
*HP0482*	hypothetical protein
*HP0483+*	*pseudo*
*HP0489*	hypothetical protein
*HP0492*	**neuraminyl-lactose binding hemagglutinin**
*HP0503*	hypothetical protein
*HP0505*	hypothetical protein
*HP0513*	hypothetical protein
*HP0519*	hypothetical protein
***HP0522***	**cag pathogenicity island protein (cag3)**
***HP0523***	**cag pathogenicity island protein (cag4)**
***HP0524***	**cag pathogenicity island protein (cag5)**
***HP0525***	**virB11 homolog**
***HP0526***	**cag pathogenicity island protein (cag6)**
***HP0527-1***	**cag pathogenicity island protein (cag7)**
***HP0528***	**cag pathogenicity island protein (cag8)**
***HP0529***	**cag pathogenicity island protein (cag9)**
***HP0530***	**cag pathogenicity island protein (cag10)**
***HP0531***	**cag pathogenicity island protein (cag11)**
***HP0532***	**cag pathogenicity island protein (cag12)**
***HP0534***	**cag pathogenicity island protein (cag13)**
***HP0535***	**cag pathogenicity island protein (cag14)**
***HP0537***	**cag pathogenicity island protein (cag16)**
***HP0538***	**cag pathogenicity island protein (cag17)**
***HP0539***	**cag pathogenicity island protein (cag18)**
***HP0540***	**cag pathogenicity island protein (cag19)**
***HP0541***	**cag pathogenicity island protein (cag20)**
***HP0542***	**cag pathogenicity island protein (cag21)**
***HP0543***	**cag pathogenicity island protein (cag22)**
***HP0544***	**cag pathogenicity island protein (cag23)**
***HP0545***	**cag pathogenicity island protein (cag24)**
***HP0547***	**cag pathogenicity island protein (cag26)**
*HP0548*	***DNA helicase***
*HP0556*	hypothetical protein
*HP0579*	hypothetical protein
*HP0583+*	hypothetical protein
***HP0584***	**flagellar switch protein (fliN)**
***HP0593***	**Adenine-specific DNA methyltransferase (mod)**
***HP0600***	**multidrug resistance protein (spaB)**
***HP0611***	**ABC transporter, ATP-binding protein**
***HP0613***	**ABC transporter, ATP-binding protein**
*HP0639+*	**putative trans-regulatory protein**
*HP0647*	hypothetical protein
***HP0663+***	**chorismate synthase (aroC)**
*HP0664+*	hypothetical protein
***HP0665+***	**oxygen-independent coproporphyrinogen III oxidase (hemN)**
***HP0666***	**anaerobic glycerol-3-phosphate dehydrogenase, subunit C (glpC)**
*HP0668+*	Remnant of type I restriction-modification polypeptide
*HP0669*	Remnant of type I restriction-modification polypeptide
*HP0670*	hypothetical protein
*HP0673*	hypothetical protein
*HP0674*	hypothetical protein
*HP0688*	hypothetical protein
***HP0700***	**diacylglycerol kinase (dgkA)**
*HP0712*	hypothetical protein
***HP0724+***	**anaerobic C4-dicarboxylate transport protein (dcuA)**
***HP0725***	**outer membrane protein**
*HP0732*	Remnant of ancestral polypeptide of unknown function
*HP0733*	Remnant of ancestral polypeptide of unknown function
*HP0734+*	hypothetical protein
***HP0737***	**putative phosphatidylglycerophosphatase A(pgpA)**
*HP0744*	*hypothetical protein*
***HP0749+***	**cell division membrane protein (ftsX)**
*HP0761*	hypothetical protein
*HP0762*	hypothetical protein
*HP0764*	Remnant of ancestral polypeptide of unknown function
*HP0766*	hypothetical protein
***HP0769***	**molybdopterin-guanine dinucleotide biosynthesis protein A (mobA)**
*HP0778+*	hypothetical protein
*HP0783*	hypothetical protein
***HP0785***	**putative outer membrane lipoprotein carrier protein**
***HP0790+***	**putative type I R-M system specificity subunit**
***HP0801+***	**molybdopterin converting factor, subunit 1 (moaD)**
***HP0808***	**holo-acp synthase (acpS)**
*HP0809*	**putative flagellar biosynthesis protein**
*HP0810*	**putative N-6 adenine methyltransferase**
*HP0812*	hypothetical protein
*HP0813*	hypothetical protein
*HP0820*	hypothetical protein
***HP0826***	**Beta-4-galactosyltransferase**
***HP0831***	**putative dephospho-CoA kinase**
***HP0845***	**thiamin phosphate pyrophosphorylase/hyroxyethylthiazole kinase (thiM)**
***HP0855***	**alginate O-acetylation protein (algI)**
*HP0856*	hypothetical protein
*HP0868*	hypothetical protein
***HP0869***	**hydrogenase expression/formation protein (hypA)**
*HP0880*	hypothetical protein
*HP0882+*	hypothetical protein
*HP0895*	hypothetical protein
*HP0897*	hypothetical protein
***HP0922-1+***	**toxin-like outer membrane protein (VacA)**
*HP0935+*	hypothetical protein
*HP0956+*	**putative ribosomal large subunit pseudouridine synthase C**
*HP0964*	**putative ATP/GTP-binding protein**
*HP0965*	**putative ATP/GTP-binding protein**
*HP0966*	hypothetical protein
*HP0982*	hypothetical protein
*HP0986*	hypothetical protein
*HP0988*	**putative IS605 transposase A**
*HP0990*	hypothetical protein
*HP0991*	hypothetical protein
*HP0994*	hypothetical protein
***HP0998***	**IS605 transposase (tnpA)**
*HP1002*	hypothetical protein
*HP1003*	hypothetical protein
*HP1004*	hypothetical protein
***HP1006***	**conjugal transfer protein (traG)**
***HP1008***	**IS200 insertion sequence from SARA17**
***HP1009***	**site-specific recombinase**
*HP1015*	hypothetical protein
***HP1045+***	**acetyl-CoA synthetase (acoE)**
*HP1051+*	hypothetical protein
*HP1074*	hypothetical protein
*HP1078+*	hypothetical protein
*HP1079*	hypothetical protein
***HP1080+***	**conserved hypothetical integral membrane protein**
*HP1081*	hypothetical protein
*HP1094*	hypothetical protein
***HP1095***	**IS605 transposase (tnpB)**
***HP1096***	**IS605 transposase (tnpA)**
***HP1115***	hypothetical protein
***HP1116***	hypothetical protein
***HP1117***	**Cysteine-rich protein X (pbp)**
***HP1121***	**Cytosine-specific DNA methyltransferase (BSP6IM)**
***HP1125***	**Peptidoglycan-associated lipoprotein precursor (omp18)**
*HP1127*	hypothetical protein
***HP1129***	**biopolymer transport protein (exbD)**
***HP1142***	hypothetical protein
***HP1146+***	hypothetical protein
*HP1149*	**putative 16s rRNA processing protein (rimM)**
***HP1164+***	**thioredoxin reductase (trxB)**
***HP1165+***	hypothetical protein
***HP1188+***	hypothetical protein
***HP1193***	**aldo-keto reductase, putative**
***HP1199***	**ribosomal protein L7/L12 (rpl7/l12)**
***HP1201***	**ribosomal protein L1 (rpl1)**
***HP1209***	**ulcer-associated gene restriction endonuclease (iceA)**
***HP1210***	**serine acetyltransferase (cysE)**
***HP1220***	**ABC transporter, ATP-binding protein (yhcG)**
*HP1221*	**putative undecaprenyl pyrophosphate synthase**
***HP1224***	**uroporphyrinogen III cosynthase (hemD)**
*HP1236*	hypothetical protein
*HP1250+*	hypothetical protein
***HP1260***	**NADH-ubiquinone oxidoreductase, NQO7 subunit (NQO7)**
*HP1283*	hypothetical protein
*HP1289*	hypothetical protein
***HP1331+***	**putative branched-chain amino acid transport protein (azlC)**
*HP1334*	hypothetical protein
*HP1351*	**HpyAIV, a type II restriction endonuclease**
***HP1352***	**Adenine-specific DNA methyltransferase (hpyAIVM)**
***HP1354***	**putative adenine-specific DNA methyltransferase**
***HP1365+***	**response regulator**
***HP1366***	**type IIS restriction enzyme R protein (MBOIIR)**
***HP1367***	**a type IIS adenosine specific DNA methyltransferase**
***HP1368+***	**a type IIS adenosine specific DNA methyltransferase**
***HP1369***	**Adenine-specific DNA methylase/** ***pseudo***
*HP1370+*	unknown
***HP1371+***	**type III restriction enzyme R protein**
*HP1382*	**putative endonuclease**
***HP1383***	**restriction modification system S subunit**
*HP1388*	hypothetical protein
*HP1390*	hypothetical protein
*HP1396*	hypothetical protein
*HP1397*	hypothetical protein
***HP1400+***	**iron(III) dicitrate transport protein (fecA)**
***HP1402***	**type I restriction enzyme R protein (hsdR)**
*HP1410*	hypothetical protein
*HP1411*	hypothetical protein
*HP1412*	hypothetical protein
***HP1415***	**tRNA delta(2)-isopentenylpyrophosphate transferase (miaA)**
*HP1424+*	hypothetical protein
*HP1426*	hypothetical protein
*HP1437*	hypothetical protein
***HP1438***	**conserved hypothetical protein**
***HP1441***	**peptidyl-prolyl cis-trans isomerase B, cyclosporin-type rotamase (ppi)**
*HP1455*	hypothetical protein
***HP1471***	**Non-functional type IIS S-subunit involved in DNA seq. specificity(BCGIB)**
*HP1502*	hypothetical protein
*HP1510*	**putative dihydroneopterin aldolase**
***HP1513+***	**selenocysteine synthase SelA, putative**
***HP1514+***	**transcription termination factor NusA (nusA)**
*HP1516+*	hypothetical protein
***HP1517-1***	**type IIS restriction enzyme R and M protein (ECO57IR)**
*HP1519*	hypothetical protein
*HP1520*	hypothetical protein
***HP1521***	**type III restriction enzyme R protein (res)**
*HP1527+*	**ComH, a periplasmic protein essential for natural competence**
***HP1534***	**IS605 transposase (tnpB)**
***HP1535***	**IS605 transposase (tnpA)**
*HP1537*	hypothetical protein
***HP1551***	conserved hypothetical secreted protein
***HP1561***	**iron(III) ABC transporter, periplasmic iron-binding protein (ceuE)**
*HP1566*	hypothetical protein
*HP1569*	hypothetical protein
*HP1570+*	**putative ABC transporter system inner membrane protein**
***HP1578***	**LPS biosynthesis protein**
*HP1587*	hypothetical protein
*HP1589*	hypothetical protein

### Cladistic analysis based on gene content of isolates, definition of gene profiles and delineation of strains

The macro-array findings concerning the presence and absence of genes were used to build bifurcating trees representing possible relationships between, or clustering of the genetic variants (Fig 1, 2, 3). Phylogenetic trees of very similar topology were found by using parsimony with various weighting schemes against gain of genes, in particular *cag*PAI, and by excluding or not genes that gave ambiguous signals. This strategy defined clusters based on significant differences in gene content (validated by high bootstrap values). Gain or losses of CDSs, as predicted by parsimony, were indicated on different parts of the phylogenetic trees (Fig 1, 2 and 3). Each strain was defined as a cluster of genetic variants sharing a same gene profile, indicated at node. The specificity of each gene profile allowed strains circulating within each family to be distinguished.

According to this cluster analysis, isolates from family D belonged to three different strains, those from family L consisted in two strains and those from family A were grouped in at least 5 strains. Strains from family D and family L differed by the presence/absence of 24 to 42 CDSs. In family A, one strain was difficult to define due to the small differences of gene profiles between neighbouring branches (corresponding to very small differences in gene content between isolates).

### Features of strains circulating within each family

**Family D**: Three different strains defined by specific gene profiles were found circulating within the family (Fig 1). Strain S1 from the father was characterized by the presence of eight CDSs coding for the transposase *IS605* (insertion sequence 605), of one CDS involved in lipopolysaccharides biosynthesis (HP1578) and the absence of a CDS coding for a DNA methyl-transferase (HP0260) (Table 1). Strain S2 from the mother differed from strain S1 by the absence of the *cag* PAI, and the presence of two CDSs associated with the *ter* plasmid (HP0433, HP0434), four CDSs belonging to the restriction/modification system (R-M system) (HP1351, HP1366; HP1367, HP1368) and one CDS coding for an alginate acetylation protein (HP0855). The two children shared the third strain (S3) characterized by the presence of four CDSs of unknown function and the absence of 24 CDSs (of which 3 belonged to the R-M system, one coding for a recombinase (HP1009) and one for a conjugal transfer protein (HP1006). The isolates from the two children differed only by two CDSs of unknown function. The strains S1 from father and S3 from children shared the same *glmM* allele G2, an allele not previously reported in any strains studied.

**Figure 4:**
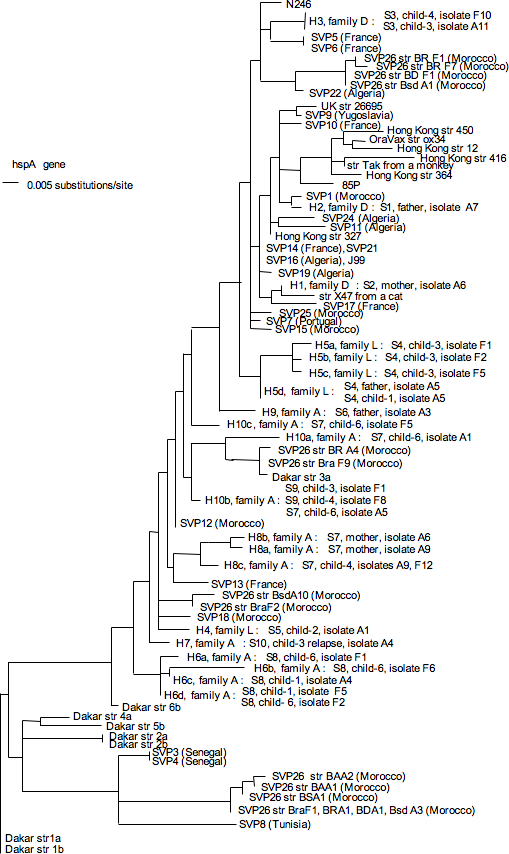
Neighbor joining unrooted dendrogram for *hspA* sequences. The scale indicates the number of substitutions per site according to the Kimura model (*see* Raymond *et al.*, 2004). Sequence names correspond to the geographic region of isolation, followed by the strain number. SVP, Saint Vincent de Paul Hôpital, Paris, France. The ethnic origin of French patients is indicated in brackets when known.

**Figure 5:**
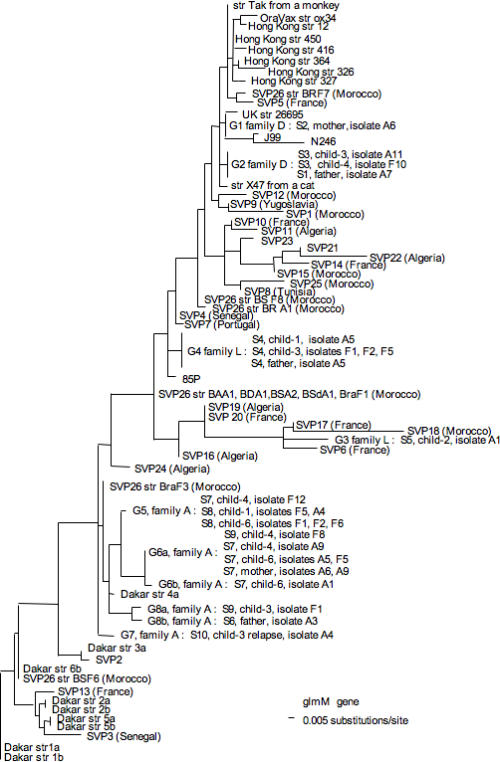
Neighbor joining unrooted dendrogram for *glmM* sequences. The scale indicates the number of substitutions per site according to the Kimura model. Sequences are named as in Figure 4.

**Family L**: Only two strains, S4 and S5, were identified (Fig 2). Strain S4 colonized the father and two children (child-1 and child-3). This cluster of isolates was validated by a bootstrap value of 93%. One of the variants of S4 from child-3 carried two additional CDSs of unknown function. Child-2 carried strain S5, differing from S4 by the presence of two IS200 and the absence of IS605. In addition, S4 and S5 harbored *hspA* and *glmM* alleles clearly different on the phylogenetic trees (Fig 4, 5). The *hspA* allele, H4 from S5 (child-2), shared close relationships with genes of strains from Africa while *hspA* alleles, H5a, H5b, H5c and H5d from S4, branched with European strains.

**Family A**: Five strains circulated within this family. Two strains, S6 and S10, were clearly different from the three others (Fig 3). S6 from the father was characterized by the absence of the *cag PAI* and the presence of 19 CDSs (including HP0855 coding for an enzyme of the R-M system, HP1578 coding for a lipopolysaccharide protein and HP1045 coding for an acetyl-CoA synthetase) (Table 1). Strain S10, isolated during the relapse of the infection in the child-3, harbored eight IS605 sequences not found in the other isolates of the family. The strains S8 (isolated from child-1 and child-6) and S9 (child-3 and child-4) belonged to distinct clusters validated by high bootstrap values. S8 was characterized by the presence or absence of 11 CDSs. The variants of S8 were distinguished by point mutations in *hspA* and *glmM* or only one to three CDSs of unknown function. The strain S9 differed from the other strains isolated in family A by the presence or absence of five CDSs. The variants of S9 from child-3 and child-4 exhibited two different allelic combinations (H10b-G8a and H10b-G6a) and differed by four CDSs coding for genes of the R-M system or of unknown function. Seven isolates from mother and two from the child-4 and the child-6 were attributed to the strain S7, despite not forming a tight cluster on the phylogenetic tree. These isolates were grouped together because their gene profiles not clearly differed with each other. They although exhibited a high diversity of allelic combinations: H8b-G6a, H8a-G6a, H8c-G6a, H8cG5a and H10b-G6a, H10a-G6b, and H10c-G6a (Fig 3). These variants of S7 differed only by CDSs belonging to the R-M system or coding for unknown functions. All except those from the mother possessed a gene coding for a conjugal transfer protein (HP1006).

### Genome dynamics and origin of strains infecting an individual

**Figure 6:**
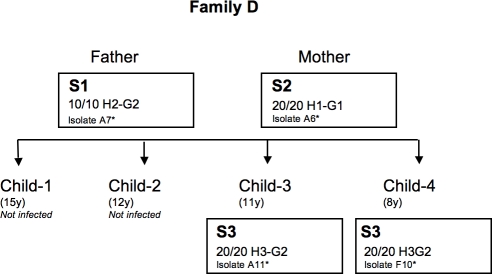
Genealogy of family D, indicating the number and the genotypes of isolates for each member. The *hspA* and *glmM* alleles are designated H and G, respectively. The alleles are numbered according to the phylogenetic cluster to which they belong (Fig 4 and 5). Lower case letters were assigned when alleles differed by point mutations. * name of the isolates studied on macroarrays. S, name of the strains defined by macro-arrays. Age of children is in brackets.

**Figure 7:**
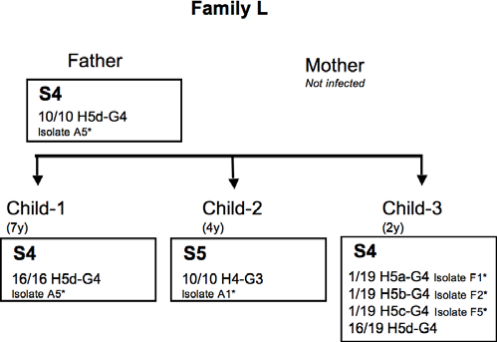
Genealogy of family L, indicating the number and the genotypes of isolates for each member. * name of the isolates studied on macro-arrays. S, name of the strains defined by macro-arrays.

**Figure 8:**
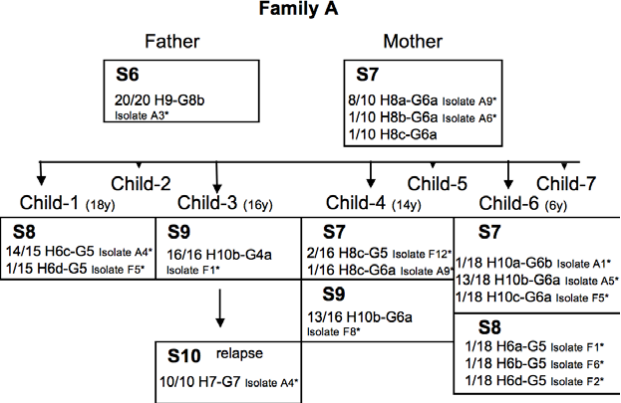
Genealogy of family A, indicating the number and the genotypes of isolates for each member. Child-2, child-5 and child-7 were not infected. * name of the isolates studied on macro-arrays. S, name of the strains defined by macro-arrays.

All individuals in the three families, except two children in family A from Morocco, were colonized by a single strain (Fig 6,7,8). Indeed, according to significant difference in their gene profiles, two different strains were identified in the stomachs of only two children (child-4 and child-6 from family A) (Fig 8).

The percentage of the tested CDSs from strain 26695 that were absent from the various isolated strains ranged from 14.8% (S1 from the family D) to 30.5% (S7 from the family A). Different isolates from a single individual differed by only point mutations, as for example in *hspA* gene (child-3, family L) or by the gain or loss of one to five CDSs (mother and child-1, family A). Gene exchange was suspected between different strains isolated for a single individual for example in child-4 of family A (Fig 3).

The phylogenetic tree based on the analysis of gene content of all the isolates studied (data not shown) showed that strains from the Moroccan family were distantly related to those from the French families.

Polymorphism was particularly evident for the three isolates from the youngest child of the family L. Twelve CDSs had signals at intermediate levels leading to different branching patterns on the phylogenetic tree based on macro-array findings (Fig 1) and *hspA* alleles differed by point mutations. In addition, one isolate (F5) differed from the others by the presence of two CDSs of unknown functions (HP1520 and HP674).

## Discussion

We studied three intrafamilial infections by *Helicobacter pylori*. Bacteria sampled at the day of endoscopy in the stomachs of the infected family members were compared. We attempted to study at least twenty isolates per patient (10 from the antrum, 10 from the fundus), which represents a high number of bacteria never used to explore *H. pylori* infections even in studies searching a mixed infection [24]–[26]. Throughout, it does not rule out the presence of minor populations with fewer organisms.

The genomic diversity of isolates was explored by macro-arrays. Such analysis is now a well-established technique for exploring the distribution of genes among clinical *H. pylori* strains [18]. However, a limitation of this approach is that point mutations, small deletions, and gene rearrangements may decrease spot intensity values leading to signals that are difficult to interpret. For these reasons, we defined gene profiles of isolates only on unambiguous signals corresponding to presence or absence of CDS. The nucleotide polymorphism of isolates, mainly point mutations, was taking into account through their *hspA* and *glmM* gene sequences. Polymorphism was particularly evident in *H. pylori* isolates from one child of family L: the bacteria had undergone minor genomic alterations, as previously described between paired of antrum and corpus isolates recovered from individual patients [27], [28]. However, using macro-arrays remains essential to recognize strains circulating in families, particularly when lateral transfers of *hspA* or *glmM* genes occurred, *i.e.* isolates H10b-G8a and H10b-G6a of S9 in family A.

### *Comprehensive overview of genomic variability of* H. pylori

A total of 134 (8.4%) of the 1590 CDSs studied were variably present in isolates from members of three different families. This percentage was high in the context of only three families, compared to the 18 to 28% of genes variably present reported using whole genome micro-array of *H. pylori* strains from different human populations [18], [20].

One class of strain-specific genes in *H. pylori* genomes is genes acquired after speciation, including those of the *cag* pathogenicity island (*cag*PAI). The chromosomal integrity of the island or the lack thereof may contribute to the progress of gastroduodenal pathology [2]. In our study, the island was absent from strains infecting the family L, variably present in strains from family D and present in all the strains, except one for the father, from family A. In isolates carrying the *cag*PAI, the 22 CDSs were found. No deletion of the *cagA*, *cagE* and *cagT* genes, involved in benign cases, was observed [29]. No link between *cag*PAI and clinical symptoms was evident from our study.

Genomes of strains differed also by the number and the nature of IS transposases, genes of the R-M system, and CDSs of unknown function. Consequences of events of acquisition or loss, the presence or absence of these genes was probably not essential for the fitness of *H. pylori*. For example, the transposable element-like sequences, *IS605*, have been reported in 31% *H. pylori* strains with copy numbers of one to nine per genome, independent of their geographical origin and of their probable virulence [30]. We found no evidence for exchange of IS between strains of *H. pylori* within families, suggesting that the strains acquired this element prior to the colonization. Most IS tested by the macro-array used are from the putative/hypothetical plasticity zone (PZ1 : HP0428 to HP0460, and PZ2: HP0982 to HP1078) of the genome 26695. All the CDSs from the PZ1 and PZ2 plasticity zones were found in both French families, but CDSs from PZ1 were absent in strains from the Moroccan family A. They are also absent from strain J99 belonging to the hspWAfrica subpopulation of hpAfrica1 population [18], [31]. The pattern of CDSs from PZ2 differed between strains from family A, but all were nevertheless closely related to that in the genome J99. Most of the genes of this region showed a GC content less than 36% indicating that they may have been imported by horizontal gene transfer from other species [19]. The acquisition (or loss) of genes in PZ2 was a major contribution to the substantial genetic diversity of strains infecting the Moroccan family (Fig 3, 8).

The number of CDSs involved in the nucleic acid metabolism (R-M system, DNA methyltransferase, endonuclease type II) varied substantially between the studied genomes as previously described [32], [33]. Most of the annotated CDSs differentiating strains within families were selfish DNA, such as that encoding restriction/modification enzymes (13/134 CDSs). Eight of them (61.54%) showed atypical dinucleotide signatures suggesting they have been acquired from divergent species [23]. The genes for R-M systems may be exchanged between strains circulating in our families as previously described for *hspA* and glmM [22], and lost because they provide no selective advantage to the organism [34]. This diversity reinforces the hypothesis of constant acquisition of new R-M systems and inactivation and deletion of the existing systems involved in DNA uptake and phage infection [35].

Of the 254 genes included on the macro-array, 147 CDSs were of unknown function and also variably present in our collection of isolates. Only 15 of the 147 (10.2%) showed atypical dinucleotide signatures [23]. As previously reported, genes of unknown function may be exchanged between strains or vertically inherited and presumably progressively lost during evolution [18].

Comparative genome analysis using macro-arrays provides insights into microbial evolution and genetic diversity in microbial populations [20], [27]. We confirmed that genes coding for R-M systems, IS transposases and many genes of unknown function are involved in the genetic diversification of *H. pylori* [20]. Some variants of the same strain differed by only two to four CDSs (1%). This observation is consistent with studies reporting that genomic contents of isolates from the same stomach may differ by 0–2% of CDSs [27], [35].

Future studies may reveal their functional status, their role in host-pathogen interactions and their importance in adaptation to genetically diverse hosts.

### Transmission of infection within families

Using macro-arrays to test for variable CDSs allowed to precise intrafamilial infection. The same strains, as assessed by micro- and macro-diversity studies, were identified in at least two members of each of the three families; this supports the accepted model that intrafamilial transmission is a major mechanism of *H. pylori* spread.

In family D, three strains were present (Fig 1, 6) and the obvious route of transmission was between siblings. However, the *glmM* allele G2, not previously found in any strains of different biogeographic origins, was present in strain S1 from the father and S3 from the two children. This indicates that recent recombination occurred between strains from the father and children, and suggests that these strains have circulated previously within the family.

The presence of strain S4 in both the father and two children of family L (Fig 2, 7) and of strain S5 only in child-2 suggested at least two different routes of infection for the children in this family: one involving intrafamilial transmission and the other route remains to be discussed (S5). Indeed, with *hspA* and *glmM* alleles similar to the sequences of African strains, conversely to S4 colonizing the father and the two other children (Fig 4, 5), S5 seems to be acquired outside of the family. However, we cannot exclude that S5 have never infected other family members (the mother?) before the day of sampling (endoscopy).

We also confirmed intrafamilial transmission between siblings in family A. Five different strains with very diverse gene contents circulated within this family. Child-4 and child-6 had mixed infections associating the strain S7 from mother with a strain (S8 or S9) also carried by an older sibling: this suggests transmission from the mother and older children to the younger children; or common sources of infection, as previously described [8], [13], [16], [36]. The oldest children were infected by strains different from those carried by their parents. These strains may have been acquired outside the family since these two children grew up far from their parents. Furthermore, after treatment of all members of this family and proved eradication, only one child was re-infected with a fully different strain. This strain isolated one year after was acquired likely outside the family, since none of the others were reinfected.

Thus, in all three families studied, two routes of acquisition may be raised: intrafamilial transmission (between parents and children or between siblings) and acquisition from outside the family.

Polymorphism was low among the strains isolated from each of the two French families, and no mixed infection was detected. Conversely, the multi-colonization of members of the Moroccan family is in agreement with data reporting that multi-colonization is more frequent in countries in which *H. pylori* infection is highly prevalent [24]–[26], [37]. The relapse of the child-3 in family A shows that re-infection from a source independent of the family, a likely event in high prevalence developing countries [38], is also possible in developed countries.

### Conclusion

Our evolutionary system-biology approach used to characterize molecular differences between isolates allowed us to document person-to-person transmission of *H. pylori* within a family. In each of these three families, the mother was not or only weakly implicated in the transmission of *H. pylori*. Macroarray analysis gives a large view of the genome diversity of *H. pylori*. This approach combined with information on the origin of the *hspA* and *glmM* alleles revealed that *Helicobacter pylori infection* may be acquired by more diverse routes than previously expected.

## Materials and Methods

### Subjects, gastric biopsies and H. pylori isolates

This study was a retrospective one, with a molecular biology analysis carried out on biopsy samples performed according to the routine handling of such patients and families in our department. All patients were investigated in a hospital setting, according to the good clinical practices, with informed consent of the endoscopic procedure followed, when applicable, by the appropriate treatment.

In this routine process, the consent for the endoscopic procedure is always written and kept in the patient's medical record. Following preliminary results, the study was presented to the local ethics committee (Comité de protection des Personnes, Ile-de-France III, Hôpital Tarnier-Cochin) which gave its approval. No extra biopsy sample or additional endoscopy was required to evaluate the *H. pylori* status of the patients. Nonetheless, prior to any endoscopic procedure, detailed information was always given to the patients or their parents in order to perform endoscopy and extensive analysis on the biopsy samples if proven *H. pylori* positive. Patients or parents gave their oral consent for this process. Noteworthy, the molecular analysis described in the paper was not anticipated at the time that the samples were taken, so that only the typing of the strains was explained to the parents.

Three families were studied. In each family, a child (index child) suffering from recurrent abdominal pain, was investigated for *H. pylori* infection. An endoscopy was performed. Biopsies from antrum and fundus were taken and cultured for *H. pylori*. After the antimicrobial susceptibility testing results, the 3 children were treated twice with a 7 day proton pump inhibitor based-triple therapy associated omeprazole, amoxicillin, and clarithromycin. The failure of *H. pylori* eradication was confirmed in each case using the ^13^C urea breath test. In the hypothesis of an intra-familial infection that may favor the persistence of the bacteria, all the parents and their siblings were tested for infection by urea breath test. When the test was positive, biopsy samples were taken from the corpus and antrum of the stomach during endoscopy, in order to obtain an antimicrobial susceptibility testing.

At all, the first family (family D) of French origin comprised two parents and three children (Fig 6). Both parents and only two children were infected (child-3 and child-4, who were 11 and 8 years old, respectively). The second family (family L) was also of French origin and consisted of two parents and three children (Fig 7). Only the father and the three children were infected (child-1, child-2 and child-3, who were 4, 4, and 2 years old, respectively). The third family (family A) originated from Morocco and included two parents and seven children (Fig 8). Both parents and four children were infected (child-1, child-3, child-4 and child-6, who were 18, 16, 14 and 6 years old, respectively). Child-2, child-5 and child-7 were not infected. Child-1 and child-3 were born in Morocco and the other children were born in France. All the infected adults suffered from gastritis and all the infected children had abdominal pain. None had received a previous anti-*H. pylori* infection treatment. All the infected members of the family, including the index child, were treated at once the same day. The eradication was controlled at least two months later by urea breath test. All subjects exhibited infection eradication. Among all the tested families, only the index child from family A, re-suffered one year later again from abdominal pain. A novel endoscopy was performed and revealed the presence of *H. pylori* infection. The parents and siblings were tested again by urea breath test and were negative.

Cultures were as previously described [22]. When possible, ten independent colonies were randomly selected from each primary culture (antrum and fundus) and sub cultured. A total of 240 isolates (80 for the family D, 55 for the family L and 105 for the family A) were independently subcultured and isolates were stored as frozen suspensions. Repetitive sequence analysis has previously found that freezing or subculturing strains had no effect on the stability of the *hspA* and *glmM* sequences.

**Nucleotide sequence accession numbers.** The sequences obtained during this study were assigned the following EMBL accession numbers: *glmM* : Family A = AM948032 to AM948040, AM948043, AM948045, AM948046, AM948049, AM948052 to AM948054, AM948057, AM948058, AM947985; Family D = AM948063, AM948064, AM948071, AM948072; Family L = AM948065, AM948067, AM948070, AM948073 to AM948075

*hspA* :Family A = AM947943 to AM947945, AM947947, AM947949, AM947950, AM947952, AM947953, AM947956 to AM947958, AM947961, AM947962, AM947964 to AM947966; Family D = AM947973, AM947974, AM947986, AM947987; Family L = AM947975, AM947976, AM947978, AM947980, AM947983, AM947988

Other sequences in the phylogenetic trees (Fig 4 and 5) were from previous studies and deposited at EMBL under accession numbers = AJ809447 to AJ809492 for *glmM* and AJ809893 to AJ810031 fro *hspA*.

### *Selection of isolates on* hspA *and* glmM *allelic variations*

A 487-bp segment, containing the 384-bp *hspA* gene (H), and a 294-bp fragment of the *glmM* gene (G) were amplified from each of the 240 isolates. Each purified PCR product was fully sequenced on both strands, using an ABI310 automated DNA sequencer (Perkin-Elmer). Each sequence obtained was positioned among 125 genes from strains of different geographic origins (Hong Kong, Senegal, Venezuela, Iran, France) in a phylogenetic tree, as previously described [22]. Sequences from different monophyletic groups were designated by their allelic name (numbered H1 to H10, for *hspA* and G1 to G8 for *glmM*) *(*An example of phylogenetic trees showing the position of different alleles is given in figures 4, 5 *).* A minus sign was added to the name of alleles sharing a same monophyletic group when sequences differed by point mutations (H1a, H1b, …).

Each isolate was named by the combination of the *hspA* and the *glmM* alleles. Analysis with these membranes allowed to identify 41 genes as being always present (ubiquitous) and 213 genes as variably present (non ubiquitous) in the genomes of 12 strains isolated in Africa, Asia and Europe and 120 strains isolated from patients with various clinical diseases (for example, H1-G1 for an isolate harboring the H1 *hspA* allele and the G1 *glmM* allele). The phylogenetic position of the alleles allowed prediction of the geographic origin of family isolates (Africa and countries affected by the slave trade, Europe and countries colonized by Europeans, or Asia). In addition, phylogenetic trees revealed that many isolates harbored recombined allele associations (one of the alleles or both being acquired by gene transfer, according to the discrepancies between their phylogenetic groups).

For the family D, three different alleles for *hspA* (designated H1, H2, H3) and two different alleles for *glmM* (designated G1, G2) formed three different allelic associations in the strains circulating within the family. For this family, four isolates were selected for macro-array analysis (Fig 6).

For the family L, five different alleles for *hspA* (H4, H5a, H5b, H5c, H5d) and two different alleles for *glmM* (G3, G4) were obtained. A total of five different allelic combinations were identified among isolates. Isolates harboring the allelic combination H5dG4 were shared by Child-1, Child-3 and the father; only two of these isolates were studied using macro-arrays. A total of six isolates from this family were studied (Fig 7).

For the family A, 12 different alleles for *hspA* (designated H6a, H6b, H6c, H6d; H7; H8a, H8b, H8c; H9, H10a, H10b, H10c) and six different alleles for *glmM* (designated G5, G6a, G6b, G7, G8a, G8b) were distinguished. A total of 14 different allelic associations were identified among the isolates from this family. Sixteen isolates, covering all the different allelic associations, were subjected to macro-array analysis: one from the father, two from the mother, two from child-1, two from child-3 (one from a relapse), three from child-4 and six from child-6 (Fig 8).

### ORF macro-array construction

Membranes commercialized by Eurogentec were used for the whole genome analysis. They consist of duplicate spots on the membranes of products corresponding to the 1590 CDSs of strain 26695, as initially described [39]. The PCR product for each CDS corresponded to the full length of the putative gene, with the exception of genes longer than 3 kilobases (kb) that were split into two or three PCR products. All together, 1637 PCR products were spotted in duplicate. Analysis with these membranes allowed to identify 41 genes as being always present (ubiquitous) and 213 genes as variably present (non ubiquitous) in the genomes of 12 strains isolated in Africa, Asia and Europe and 120 strains isolated from patients with various clinical diseases (data not shown).

For the in house nylon membranes, 296 PCR products were amplified in four 96-well microtiter plates; they correspond to 41 ubiquitous (some of which were spotted more than once) and 213 distinct non-ubiquitous genes of the genome of strain 26695 (Table 1). Amplification reactions were performed in 2× 100 µl reaction volume using as a template 2 µl of DNA corresponding to the recombinant plasmid containing the full length CDS inserted into the pILL570 derivative vector. Amplicons were purified on multiscreen PCR plates (Millipore Manu 3050, Saint Quentin, France). The concentration and size of each PCR product were verified on agarose gels; gene identity was definitively assigned following the sequencing of 300 base pairs (bp) of each PCR product. The 384 samples were then transferred from the four 96-well plates to a single 384-microtiter plate using a robot (Hydra). Each PCR product was spotted in triplicate on a nylon membrane (Qfilter, Genetix 22.2×22.2 cm, N+) using a Qpix robot (Genetix). Denatured 26695 genomic DNA was spotted in triplicate at the four corners of the membrane (positive controls) and 7 squares were left empty as negative controls. Following spot deposition, membranes were fixed for 15 min in 0.5 M NaOH-1.5M NaCl, washed briefly in distilled water, and stored wet at −20°C until use. Each membrane was used once.

### Macro-array experiments

Chromosomal DNA was prepared using the Qiamp (Qiagen, Courtaboeuf, France) technique. Aliquots of 250 µl of DNA were sonicated for 20 s at 50% cycle, setting 3, in a Branson sonicator 450. The adequacy of the fragment size was assessed on 0.7% agarose gels before radiolabelling. Aliquots of 25 to 50 ng of sonicated DNA were adjusted to a volume of 10 µl, heat-denatured for 5 min at 100°C, then cooled immediately on ice. They were labelled by random priming with 2 µl of ^33^P-dCTP in a reaction mixture (20 µl) containing 11.5 µl of LS buffer (25 volumes of 1 M HEPES, pH 6.6, and 25 volumes of DTM buffer, containing 100 µM of each dATP, dGTP and dTTP, prepared in 250 mM Tris-HCL, pH8, 25 mM MgCl2, 0.36% β-mercapto-ethanol) and 1 µl BSA (10 mg ml^−1^) and 0.5 µl of Klenow DNA polymerase (Amersham Pharmacia Biotec, Orsay, France). Labelling was performed for 3 h at room temperature. Unincorporated radionucleotides were removed by purification on Quick Spin Sephadex G-25 columns (Roche Diagnostics, Meylan, France). Immediately before use for hybridization, the sonicated, labeled, and purified chromosomal DNA was heat-denatured and cooled on ice.

Commercialized membranes from Eurogentec with 590 CDSs of strain 26695 and home-made membranes harboring a subset of genes (213 non-ubiquitous and 41 ubiquitous) in triplicate were used. Both of these membranes carry PCR products that matched entire genes as established for strain 26695. Each nylon membrane was re-hydrated for 15 min in 15 ml 2×SSC buffer (300 mM NaCl, 30 mM trisodium citrate 2H_2_, pH 7), and pre-hybridized for 2 hours at 65°C in 15 ml hybridization buffer (5×SSC, 2% SDS, 1×Denhardt's solution, 0.02% Ficoll type 4 (Sigma, Saint Quentin Fallavier, France), 0.02% polyvinyl-pyrolidone (Sigma), 0.02% BSA and 0.01% salmon sperm). Hybridization was conducted in 5 ml pre-warmed (65°C) hybridization mixes containing the heat-denatured probe with overnight incubation at 65°C under rotary agitation. Membranes were washed once at room temperature in 100 ml 0.5× SSC, 0.1% SDS, the four times (20 min each) at 65°C in 50 ml of the same buffer. Membranes were sealed in polypropylene bags and exposed for 25 hrs to a Phosphoimager screen (Molecular Dynamics).

Screens were scanned on a Storm 860 machine (Molecular Dynamics). Image analysis and quantification of hybridization intensities for each spot were performed using the Xdots Reader program (COSE) and determined in pixels. A) For the whole genome DNA array membrane (Eurogentec membranes), the average intensity of the empty spots was subtracted from each spot intensity value. This value was then normalized by dividing it by the average of all significant intensity values on each filter. For ratio calculations, a reference array was used, and was built by combining the average normalized data from 10 independent labelling and hybridization experiments with the genomic DNA of *H. pylori* strain 26695 to the Eurogentec. B) For home-made arrays, the intensity of the background surrounding each spot was subtracted from that of each of the spots. Twenty-one homologous hybridizations were performed and were used for normalization. The average intensity of the 41 ubiquitous genes was calculated for each reference array. This number served to allocate a reference array to each heterologous hybridization (average of the ubiquitous spots from the heterologous and the homologous reference hybridizations were not significantly different, Student's t test) and to calculate the ratio used for normalization.

### Determination of Gene Content of Isolates and Data analysis

Following normalization, the data were analyzed by attributing a binary score (presence/absence). To define the cutoff ratio for the presence of a gene, we analyzed the results for the sequenced *H. pylori* J99 DNA hybridized with *H. pylori* 26695. The threshold for the absence of a gene was defined as <0.25. A predictor for presence of genes based on median hybridization ratios and standard deviation of the ubiquitous genes on the 21+16 membranes was established to determine positive cut-off values. A ratio cut-off for all data sets was obtained at a level >0.36. Ratios from 0.26 to 0.35 were considered as indicating an uncertain or uninterpretable signal.

The final data set consisting of three different values (0 = absent, 1 = uncertain, 2 = present) was incorporated into the PAUP40b4 software program [40]. Phylogenetic trees of the *Helicobacter pylori* isolates were generated using different assumptions for parsimony analysis, including equal weighting or 10∶1 weighting against acquisition of the pathogenicity islands (to reduce the number of insertion-deletion events for this region). The confidence level at each node was evaluated by bootstrap analysis (1000 bootstraps). Clusters of isolates with bootstrap values up to 90% were used to define limit of strains.

A list of genes predicted to be different at each node was obtained by parsimony analysis using equal weighted characters and the Branch and Bound algorithm. Description of strains, presented in the results section, only includes the presence or absence of gene such that the level of signal was 2 or 0 (uncertain values were not taken into account).
